# Prioritization of family member sequencing for the detection of rare variants

**DOI:** 10.1186/s12919-016-0035-8

**Published:** 2016-10-18

**Authors:** Rachel Sippy, Jill M Kolesar, Burcu F Darst, Corinne D Engelman

**Affiliations:** 1 Department of Population Health Sciences, University of Wisconsin-Madison, 610 WARF Building, Madison, WI 53726 USA; 2 School of Pharmacy, University of Wisconsin-Madison, 777 Highland Avenue, Madison, WI 53705 USA

## Abstract

**Background:**

The advent of affordable sequencing has enabled researchers to discover many variants contributing to disease, including rare variants. There are methods for determining the most informative individuals for sequencing, but the application of these methods is more complex when working with families. Sets of large families can be beneficial in finding rare variants, but it may be unfeasible to sequence all members of these family sets.

**Methods:**

Using simulated data from the Genetic Analysis Workshop 19, we apply multiple regression to identify cases and controls. To find the best controls for each case, we used kinship coefficients to match within families. Selected cases and controls were analyzed for rare variants, collapsed by gene, associated with hypertension using the family-based rare variant association test (FARVAT).

**Results:**

The gene with the strongest simulated effect, *MAP4*, did not meet the Bonferroni corrected significance threshold. However, analysis of cases and controls using our selection method substantially improved the significance of *MAP4*, despite the reduction in sample size.

**Conclusions:**

Taking the additional steps to select the optimal cases and controls from large family data sets can help ensure that only informative individuals are included in analysis and may improve the ability to detect rare variants.

## Background

Whole-genome sequencing (WGS) is an important tool in the discovery of rare variants that influence disease. Family-based association studies have likewise been crucial in the fine-mapping of genetic variants contributing to complex disease. Decreased sequencing costs have made it increasingly feasible to sequence large families or even large sets of families, but WGS remains too expensive for most studies. To address this, a subset of family members may be selected for WGS, but it can be difficult to determine which configuration of family members will have the greatest power to detect rare variants. Extreme phenotyping is an approach that compares individuals at opposite ends of the phenotypic spectrum with the thought that rare causal variants will be enriched for in the extremes of complex traits [1–3]. The appeal of this approach is its cost-effectiveness; however, the decrease in cost relies on the ability to cheaply phenotype many more patients than will be sequenced [3]. A drawback is the decreased sample size, which can result in loss of power. We modified the process of extreme phenotyping and combined it with family-based selection to make the best use of the data. We defined our cases and controls as individuals with extremes of unexplained variation in systolic blood pressure (SBP) after adjusting for covariates in a regression analysis; these individuals are most likely to have a genetic component explaining their SBP [1, 4]. As a second step, we used kinship coefficients to eliminate those individuals who are least likely to contribute useful genetic information to the analysis because they are either too closely related (eg, parent–child) or unrelated.

## Methods

### Study population

We analyzed replicate 1 of the simulated data set from the Genetic Analysis Workshop 19 (GAW19) T2D-GENES Project 2, a family data set with WGS data [5], with knowledge of the simulation model. The provided data set consisted of family-based WGS data and simulated phenotypes for diastolic blood pressure (DBP) and SBP. Covariates included sex, age, hypertensive status, antihypertensive medication use, and smoking status. Prior to modeling, families without sequencing data available for any family member were omitted. The remaining sample consisted of 261 individuals with hypertension and 458 individuals without (base cases and controls; Table 1).

**Table 1:** Descriptive characteristics of base population, potential cases and controls, and selected cases and controls

	Base cases (*n* = 261)	Base controls (*n* = 458)	Potential cases (*n* = 170)	Potential controls (*n* = 277)	Selected cases (*n* = 128)	Selected controls (*n* = 188)	Selected cases vs. controls
Genes excluded	42		39		38		NA
Gene sets	1389		1377		1345		NA
Age (years)	52.4 (17.3)	33.0 (13.6)	49.5 (17.0)	35.2 (14.8)	49.1 (17.4)	35.8 (14.9)	<0.0001
	16.1–99.0	11.1–83.0	16.1–90.3	12.1–83.0	16.1–85.0	16.0–83.0	
SBP (mm Hg)	143 (9.5)	116 (13.0)	146 (8.7)	109 (11.7)	146 (8.7)	110 (11.4)	<0.0001
	102–186	72–140	123–186	72–139	123–186	72–139	
DBP (mm Hg)	78 (9.9)	70 (8.7)	80 (9.3)	68 (8.5)	80 (9.3)	69 (8.1)	<0.0001
	49–102	46–89	54–102	46–87	54–102	48–87	
Males	117 (45)	194 (42)	70 (41)	112 (40)	56 (44)	75 (40)	NS
Smokers	54 (21)	97 (21)	35 (21)	59 (21)	30 (23)	46 (25)	NS

*DBP* diastolic blood pressure, *NA* not applicable, *NS* not significant, *SBP* systolic blood pressure

Data are presented as mean (standard deviation) and range, n (%), or *p* values

Mean values were compared using a *t*-test; proportions were compared with a chi-squared test

### Extremes of unexplained variation

**Fig. 1:**
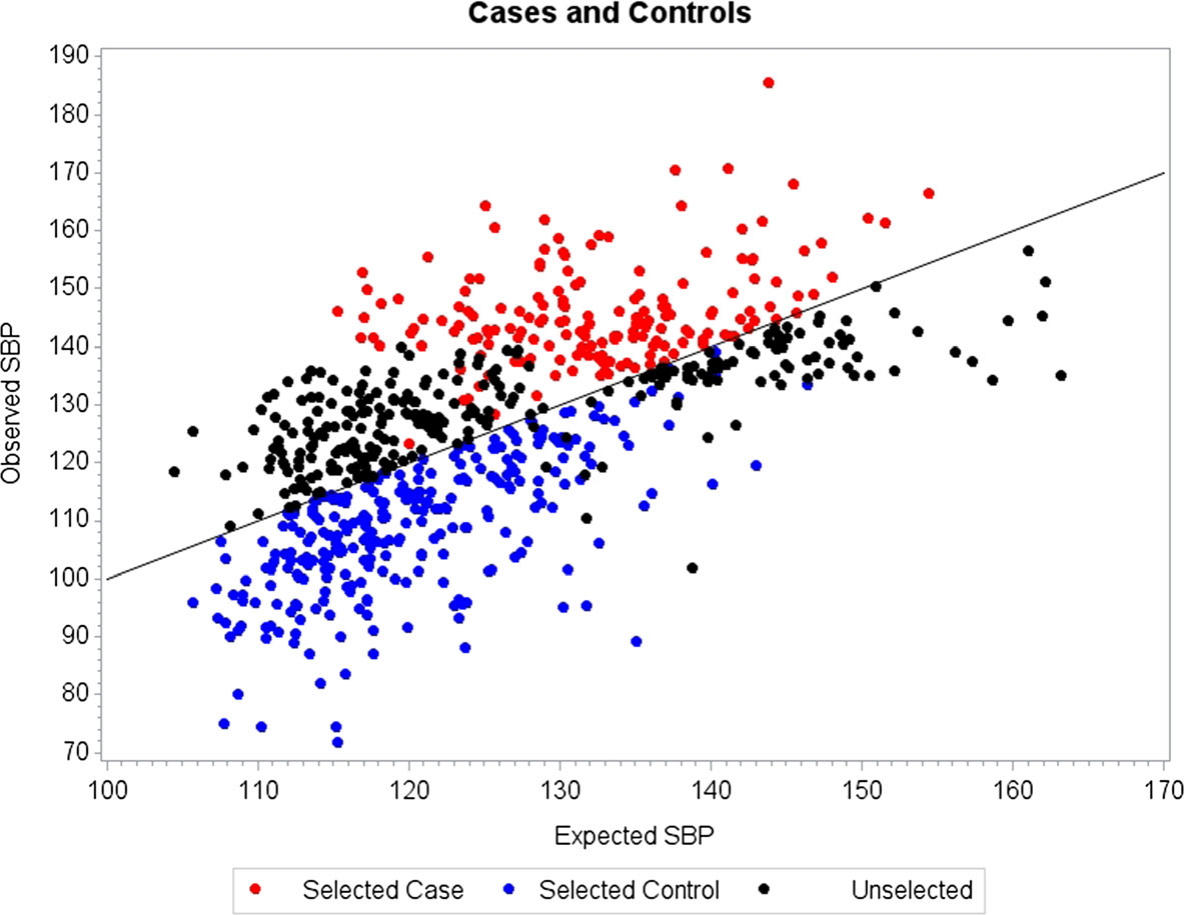
Modeling for selection of cases and controls. The base population used in modeling (*n* = 719) were plotted with their observed systolic blood pressure (SBP) and their expected SBP as predicted by multiple regression. Subjects in red are hypertensive above the mean and were designated as cases (*n* = 170). Subjects in blue are nonhypertensive below the mean and were designated as controls (*n* = 277)

To define cases and controls, we modified an approach that selects participants with variation in their phenotype that is unexplained by known nongenetic risk factors, and thus are most likely to have a genetic component [1, 4]. Using SBP as the outcome, we used multiple regression to adjust for the following nongenetic variables that affect SBP: age, sex, smoking status, and antihypertensive medication use. The original data were longitudinal; for subjects with hypertension, the first year with this diagnosis was used in the model. If the year used had missing data and the next year had more complete data, that next year was used. For those without hypertension, the year with the most complete data was used. Subjects with hypertension who were above the regression line were those with unexplained high SBP and were selected as potential cases (*n* = 170; see Table 1). These cases are identified in red in Fig. 1. Subjects without hypertension who were below the regression line were those with unexplained low SBP and were selected as potential controls (*n* = 277; see Table 1). These potential controls are identified in blue in Fig. 1.

### Prioritization of subjects

**Fig. 2:**
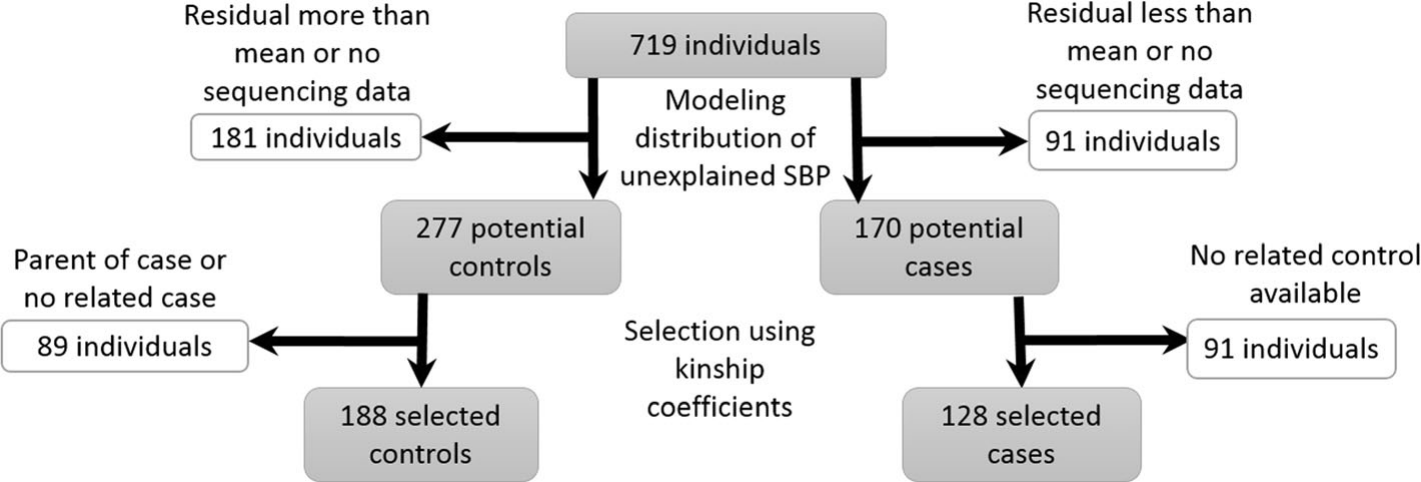
Selection of cases and controls. Multistep process using modeling to choose potential cases and controls, and kinship coefficients to select cases and controls

The process for control selection is outlined in Fig. 2. Modeling resulted in several controls being available for each case; however, the familial relationship between these potential controls and cases had not yet been taken into consideration. Family structure was determined by kinship coefficients calculated with the family-based rare variant association test (FARVAT) using pedigree data [6]. Controls who were unrelated to any case were excluded, as they were genetically uninformative. In addition, parent–child pairs may be less powerful in association analyses as a result of overmatching [7], so controls who were parents of cases were excluded. Only nonparent controls who were related to cases (ie, with a nonzero kinship coefficient) were included in the analysis, and any cases without a related control were excluded. This resulted in some cases with multiple controls, and in other cases with only a single control.

### Quality control of sequencing data

In addition to the quality control (QC) performed by the organizers of Genetic Analysis Workshop prior to release [5], further QC steps were taken using VCFtools version 0.1.12a [8] for chromosome 3, which initially included 1,757,452 sites among 464 sequenced individuals. No individuals were missing more than 10 % of calls, and thus, none were removed. Sites with a call rate of less than 95 % were removed (210,954 sites), as were sites that were out of Hardy-Weinberg equilibrium within the founders (6903 sites removed using *n* = 91 founders) at a *p* value cutoff of less than 2.9 × 10^−8^ (Bonferroni corrected: 0.05/1,546,498 = 3.2 × 10^−8^) leaving a total of 1,539,595 sites. Sites that did not pass QC were then removed from the data set of imputed genotypes that included 959 subjects (both sequenced individuals and those with imputed genotypes using the 464 sequenced subjects as input for the imputation). This data set contained 1,215,399 imputed sites, of which 87,555 sites were removed as a result of the aforementioned QC process, leaving 1,127,844 sites for analysis.

### Annotations

Gene-based annotation was performed with the sites remaining after QC using ANNOVAR (Annotate Variation) [9] and the human genome RefSeq database based on hg19. Sites in intragenic regions or outside of a gene were mapped to the closest gene. Those that were further than 5 kbp from a gene were excluded, as the simulation model selected causal variants that were within this range, which left 566,962 sites (560,882 out of range).

### Genetic analysis

Sequencing data from chromosome 3 for each set of cases and controls (base, potential, and selected) were analyzed using FARVAT [10]. FARVAT allows for the use of a dichotomous outcome and takes little computational time. FARVAT provides burden-, variance component–, and SKAT-O–type tests, and additionally provides the Pedigree Combined Multivariate and Collapsing (PedCMC) [11] and collapsing-based tests [12]. We utilized the variance component-type test as this test performs well for genes with functional rare variants having effects in the opposite direction, as is likely to be the case for most genes [6]. Users have the option to specify an offset to improve statistical efficiency. We chose the disease prevalence-based offset, using the hypertension prevalence of 0.26 among Hispanic adults as reported by the National Health and Nutritional Examination Surveys (NHANES) [13]. In addition, age and sex were included as covariates.

## Results

**Fig. 3:**
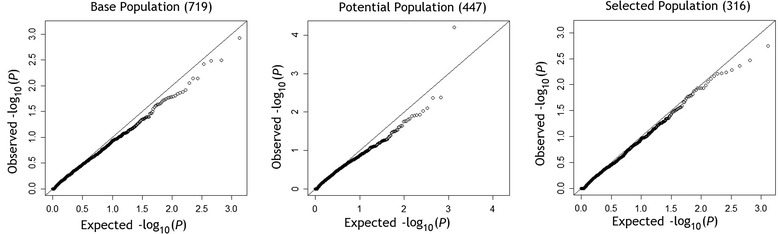
Quantile-quantile (Q-Q) plots of analyses. Q-Q plots of each analysis, including base population, potential cases and controls, and selected cases and controls

Table 1 provides descriptive results of potential and selected cases and controls. Gene sets on chromosome 3 were analyzed by FARVAT; some gene sets were excluded, as FARVAT will not analyze gene sets with only 1 single nucleotide polymorphism (SNP). FARVAT recalculates minor allele frequency among each set of individuals being analyzed, resulting in a different number of gene sets for each set of cases and controls, as shown in Table 1. After Bonferroni correction for multiple testing (*p* = 0.05/1389 = 0.000036), none of the genes reached significance for any of the 3 sets of cases and controls. Because *MAP4* was simulated to be significantly associated with SBP, Table 2 includes the results for *MAP4*, along with the 10 most significant genes for each analysis, which tended to vary. Of the genes on chromosome 3, only *MAP4*, *FLNB*, and *ABTB1* were simulated to have an effect on SBP, explaining 7.79 %, 0.29 %, and 0.13 % of the total variance in SBP. Although *MAP4* did not meet the Bonferroni-corrected significance threshold, analysis of potential cases and controls showed improved significance for *MAP4* over the analysis of all individuals in the base population, and analysis of selected cases and selected controls further improved the significance of *MAP4*. Figure 3 displays quantile-quantile plots of each analysis; these plots show no inflation of the observed *p* values, indicating that type I error was controlled.

**Table 2:** Analysis of genes associated with hypertension in simulated data

	*Base cases & controls* (*n* = *719*)		*Potential cases & controls* (*n* = *447*)			*Selected cases & controls* (*n* = *316*)		
*Rank*	Gene	*p* Value	Rank	Gene	*p* Value	Rank	Gene	*p* Value
*1*	*PAQR9*-*AS1*	0.0011739	1	*MIR4790*	0.0000621	1	*CHMP2B*	0.0017687
*2*	*CISH*	0.0031990	2	*PAQR9*-*AS1*	0.0041593	2	*CSPG5*	0.0033962
*3*	*MIR4790*	0.0032477	3	*RUVBL1*-*AS1*	0.0043295	3	*SEMA3B*	0.0043256
*4*	*TMIE*	0.0038134	4	*SPSB4*	0.0077963	4	*FGD5*-*AS1*	0.0051885
*5*	*ERICH6*-*AS1*	0.0071538	5	*SEMA3B*	0.0093311	5	*DHX30*	0.0058120
*6*	*LOC102724699*	0.0071575	6	*MBNL1*	0.0118256	6	*SEC22C*	0.0060621
*7*	*DPPA2P3*	0.0089600	7	*ERICH6*-*AS1*	0.0121301	7	*ATRIP*	0.0061709
*8*	*IMPDH2*	0.0121196	8	*CIDEC*	0.0123527	**8**	***MAP4***	**0.0067952**
*9*	*MAP6D1*	0.0131694	9	*PLXNB1*	0.0128340	9	*DLG1*-*AS1*	0.0077826
*10*	*IGSF10*	0.0138335	10	*FGD5*-*AS1*	0.0152014	10	*CHMP2B*	0.0086741
***388***	***MAP4***	**0.3111750**	**262**	***MAP4***	0.2198460			

Analysis of genes on chromosome 3 among different sets of cases and controls. *MAP4* is shown in bold, as it has the strongest simulated effect on systolic blood pressure

## Discussion

The potential power of family data is appealing for the discovery of rare variants that contribute to complex disease. Family data sets can contain hundreds or thousands of individuals, and WGS may not be feasible for every individual in every family. Frequently, researchers will select some family members for sequencing and then impute sequencing data for the remaining family members using existing genome-wide SNP data, however, this can still be costly and the accuracy of imputation varies depending on the approach used [14, 15]. As an alternative, it is possible to limit analyses to fewer family members yet bypass imputation. Careful selection of cases and controls is key to narrow the potential candidates for sequencing. Our multistep approach can be applied to any outcome and allows elimination of those individuals who are least likely to have a genetic component to their outcome, with further elimination of those individuals who will be genetically uninformative to a rare variant association analysis. Multiple factors contribute to complex disease, and it may be important to consider all of these factors in the effort to find genetic determinants. By using multiple regression, we were able to take several covariates into consideration; each of these covariate phenotypes is easily and inexpensively obtained. The inclusion of these covariates allowed us to focus our attention on those individuals with unexplained and, likely, genetic hypertension. Through this approach, cases and controls were not simply defined as those with the highest and lowest blood pressures, respectively, but rather those with blood pressure that is higher or lower than expected given their age, sex, smoking habits, and blood pressure medication usage. The use of theoretical kinship coefficients ensured only genetically informative individuals were included in the analysis. As with any selection process, the sample size decreased as the requirements for inclusion became more stringent. While this decrease reduces costs, loss of power from decreased sample size is a serious concern. In addition, the combination of multiple phenotypic components into a case definition forces the use of a dichotomous outcome during analysis, which generally results in a loss of power. However, we found that the signal for *MAP4*, the gene with the strongest simulated effect on SBP, improved with each step of the selection process, indicating that our selection process overcame the loss of power because of a decrease in sample size and dichotomization of a quantitative trait.

## Conclusions

Family data can be useful for the detection of rare variants, but must be carefully analyzed. There are options to prioritize the selection of cases and controls for sequencing and analysis. Careful case definitions, combined with information on family structure, can help ensure that only the most informative individuals are chosen for sequencing. This can help keep costs low and, potentially, improve the ability to detect rare variants. However, loss of power is a real concern, meaning the selection process may only yield meaningful results if there is a large base population from which to select.
